# The Formation of Character

**Published:** 1879-06

**Authors:** 


### THE FORMATION OF CHARACTER.

There is a practical as well as a scientific basis for the position taken by the Rev. Phillips Brooks in a recent discourse in this city, namely, that the law of evolution rules in the moral as well as in the physical world. Nature does not create, but is always developing. In last summer's roots nature finds the germ for next summer's verdure.

"If somebody should give me a diamond to carry to Europe, I can know exactly how much would be lost to the world were I to drop it into the sea; but if a seed should be given me, I can only regard it with awe as containing concealed within it the food of untold generations. That is the difference between looking at truth as a diamond or as a seed—as final or germinal.

"In all training of character, continuity and economy must be supreme. The notion that character is spontaneous is held by most people in the earlier portion of their lives, and is wrong. When they discover this, nine-tenths change to the other extreme. This is wrong too. Hosts of young men think that their character will form of itself and that they will necessarily become better as they grow older. Hosts of old men believe that their character is fixed and that it is impossible for them to become better. Such beliefs are foolish. People are also wrong in thinking that they can put off their bad traits and put on good traits. The old failures can not be thus transformed, but out of the old habits new can be formed. This is what many a poor creature needs to know. We must make what we are to be out of what we are already."—*Scientific American.*

